# A user-friendly clinical practice guideline summary for managing low back pain in South Africa

**DOI:** 10.4102/sajp.v76i1.1366

**Published:** 2020-02-20

**Authors:** Jessica Stander, Karen Grimmer, Yolandi Brink

**Affiliations:** 1 Division of Physiotherapy, Faculty of Medicine and Health Sciences, Stellenbosch University, Cape Town, South Africa

**Keywords:** physiotherapy, clinical practice guidelines, knowledge translation, low back pain, evidence-based practice

## Abstract

**Background:**

Clinical practice guidelines (CPGs) provide conveniently packaged evidence-based recommendations to inform clinical decisions. However, intended end-users often do not know how to source, appraise, interpret or choose among CPGs. Moreover, it can be confusing when recommendations on the same topic differ among CPGs, in wording, intent and underpinning evidence.

**Objectives:**

This article reports on the processes of: (1) identifying current CPGs for acute and subacute low back pain (LBP) to fit the needs of South African physiotherapists, (2) collating and summarising CPG recommendations to produce a user-friendly end-user product and (3) testing the utility of the summary CPG document on South African physiotherapy clinicians to efficiently determine acceptability, appropriateness and feasibility to inform clinical decision-making.

**Method:**

An adapted approach was followed by systematically searching online CPG repositories and online databases for LBP CPGs; screening and critically appraising identified CPGs; summarising recommendations from relevant CPGs and organising them into clinical practice activities. Feedback on utility was obtained from 11 physiotherapists.

**Results:**

Three high-quality, international CPGs provided 25 recommendations on the assessment and management of acute and subacute LBP relevant to South African physiotherapy practice. They were organised into 10 headings. Physiotherapy user feedback suggested that this document would assist in clinical decision-making.

**Conclusion:**

Organised recommendations extracted from multiple, relevant CPGs provide an end-user-friendly resource for physiotherapists treating LBP.

**Clinical implications:**

Collated and organised CPG recommendations may effectively assist South African physiotherapists’ clinical decision-making in assessing and managing patients with acute and subacute LBP.

## Background

Clinical practice guidelines (CPGs) are ‘a convenient way of packaging evidence and presenting recommendations to healthcare decision makers’ (p. 6) (Treweek et al. 2013). Recommendations in CPGs may assist physiotherapists in making evidence-informed clinical decisions (Louw et al. 2018). Low back pain (LBP) is a leading cause of disability in both high-income and low- to middle-income countries, being rated as the fourth leading cause of disability-adjusted life years (Hurwitz et al. 2018). A recent systematic review found that both the point and annual prevalence of LBP were higher among African populations compared to global LBP prevalence (Morris et al. 2018). This may be attributed to heavy physical work and manual lifting, sustained flexion postures and psychosocial factors (including fear-avoidance beliefs, catastrophising, anxiety and illness perception) (Igwesi-Chidobe et al. 2017; Tella et al. 2013).

Adherence to LBP CPGs may decrease health care utilisation, improve treatment efficacy and decrease health care costs (Hanney et al. 2016). However, Basson (2011) found that there was a mismatch between CPG recommendations and the current South African physiotherapy practice in the management of LBP (Basson 2011). Even though respondents reported using some interventions for which there was convincing evidence, they also reported using many interventions with little or no evidence of effectiveness (Basson 2011). More recently, these findings were echoed in international studies involving Swedish, US and Brazilian physiotherapists (Bernhardsson et al. 2015; De Souza, Ladeira & Costa 2017; Ladeira, Cheng & Hill 2015). Reported barriers to South African physiotherapists’ uptake of CPGs include resource constraints (i.e. lack of time, high workload, lack of financial remuneration), lack of knowledge and skills in CPG utilisation and limited organisational support (Stander, Grimmer & Brink 2019). These barriers are similar to international factors influencing CPG uptake (Da Silva et al. 2015; Scurlock-Evans, Upton & Upton 2014) but are magnified by South African health care service delivery challenges such as high ratio of patients to physiotherapists, environmental factors, cost and knowledge barriers for patients to access physiotherapy care and physiotherapists having variable access to evidence (Stander et al. 2019).

If the current, high-quality CPG recommendations are presented in an easier-to-use format and are more readily accessible for busy clinicians, this may improve CPG uptake in clinical practice (Machingaidze et al. 2018; Stander et al. 2019). Furthermore, the recommendations need to be relevant to local contexts; thus, in South Africa, recommendations should take into consideration the diversity of South African health care, and the variable ways in which physiotherapy services are provided (Dizon et al. 2017, 2018; Kredo et al. 2018). The South African Guidelines Excellence (SAGE) project provides tools to develop, adapt, adopt, contextualise and implement primary care CPGs for low- to middle-income countries (Machingaidze et al. 2015). The project has also developed a three-tier model for the efficient production of quality, contextually relevant CPGs (Machingaidze et al. 2018). This model explains not only essential CPG writing steps (Tier 1 and 2) but also the importance of end-user-friendly ‘how to do it’ documents (tier 3) to disseminate evidence to end-users. Tier 1 (body of evidence) and Tier 2 (expert input and consultation processes) address the processes whereby CPGs are developed through systematic, rigorous and methodologically sound processes. Schünemann et al. (2014) presented the Guidelines 2.0 checklist as a guideline to complete these processes (Schünemann et al. 2014).

Item 16 in Guidelines 2.0 is about ‘Dissemination and implementation: Focusses on strategies to make relevant groups aware of the guidelines and to enhance their uptake (e.g. publications and tools such as mobile applications)’. This essentially refers to Tier 3 documents (‘shorter, simpler, more concise and user-friendly guidance documents’ for a target audience (i.e. clinicians, patients) as described by Machingaidze et al. (2018:9). However, there is minimal information about how best to present recommendations in ways that are relevant for, and acceptable to, end-users.

In resource-constrained environments, *de novo* development of CPGs using the steps outlined in Guidelines 2.0 may not be practical, feasible or cost-effective. To the authors’ knowledge, there is no South African CPG for the management of acute or subacute LBP. In lieu of writing a *de novo* CPG for LBP management for South African physiotherapists, which included contextualised Tier 3 documents, we explored how currently available, well-written CPGs might be adapted, adopted or contextualised to guide LBP management for physiotherapists working in South African health care environments (Dizon, Machingaidze & Grimmer 2016; McCaul et al. 2018).

This article reports on the processes of:

- identifying current CPGs for acute and subacute LBP to fit the needs of the South African physiotherapists
- collating and summarising CPG recommendations to produce a user-friendly end-user document
- testing the utility of the summary CPG document on South African physiotherapy clinicians to determine acceptability, appropriateness and feasibility to efficiently inform clinical decision-making processes.

## Methods

The approach taken to develop the user friendly summary are described below:

Step 1: The approach described by Gonzalez-Suarez, Dizon & Grimmer-Somers (2012) was followed:

- *Systematically searching for LBP CPGs*: A search for acute and subacute LBP CPGs was conducted on the websites of reputable guideline developers (Scottish Intercollegiate Guidelines Network, New Zealand Guidelines Group, NHMRC, National Institute for Clinical Excellence), the National Guideline Clearinghouse site and the www.google.com search engine, from inception to January 2019. Nine electronic databases (CINAHL, BIOMED CENTRAL, Cochrane, PEDro, PROQUEST, PUBMED, OTseeker, Scopus, ERIC) were also searched from inception to January 2019, to ensure that all eligible CPGs were identified. Search terms and variations thereof used included: ‘clinical practice guidelines AND (acute LBP OR subacute LBP)’. Table 1 outlines an example of the search strategies used. Only CPGs published in English and available in full-text format were included. Clinical practice guidelines were excluded if they were only available in abstract format.
- *Screening CPGs*: Potentially relevant CPGs were screened for scope and purpose relating to physiotherapy assessment and management of adults suffering from acute or subacute LBP. It was essential to determine when selecting CPGs that their purpose clearly linked with the needs of their end-users (physiotherapists working in any South African health care environment); their scope included relevant clinical question(s), and they incorporated the most current and the best available evidence (Dizon et al. 2016; Machingaidze et al. 2015). Thus, CPGs written in the last 5 years were specifically sought as being the most likely to include current evidence. The screening process also considered South African physiotherapists’ requirements of CPGs and their recommendations, as determined from interviews with private, public and educator physiotherapists (Stander et al. 2019). These criteria included: ease of use; ease of accessibility; low-to-no cost of accessing CPG; and relevance in, and applicability to a local South African context (Stander et al. 2019).
- *Critically appraise relevant CPGs:* The Appraisal of Guideline ResEarch and Evaluation (AGREE II) Checklist was used to critically appraise the methodological quality of potentially relevant CPGs (Brouwers et al. 2010). The instrument seeks information on guideline scope and practice, quality, clarity of presentation, currency, rigour of development and stakeholder involvement. The AGREE II provides domain scores, not an overall score. However, the requirement of a minimum of two appraisers for the AGREE II appraisal ensures better reliability of the score (Brouwers et al. 2010).

**TABLE 1:** Search strategy example.

Number	Search strings
1	((‘Practice Guidelines as Topic’[Mesh]) AND ‘Practice Guideline’ [Publication Type]) AND ‘Low Back Pain’[Majr]
2	(‘clinical practice guidelines’ OR ‘guidelines’) AND ‘Low Back Pain’ [Majr]

Step 2: All relevant recommendations from all included CPGs were extracted verbatim. The strength of the body of evidence for each recommendation was not included because of the different ways of reporting the strength of recommendations. The recommendations were categorised according to clinical purpose and organised to fit on a two-sided A4 page (for ease of use in clinical settings) (Machingaidze et al. 2018). The recommendations were organised under headings of assessment; red flags; imaging; management; return to work; advice and education; exercise and electrophysical modalities. The recommendations within each heading were then clustered according to the wording and focus of each recommendation (Gupta et al. 2016; Hussain, Michel & Shiffman 2009; Shiffman et al. 2005). No attempt was made to synthesise the original recommendations into composite recommendations (Grimmer et al. 2019). By keeping the recommendations separate, the authors believe that the Tier 3 document provides clinicians with clear and easy-to-follow guidance from the parent CPGs to assist in their clinical decision-making.

A summary statement was compiled for each cluster of recommendations. The summary statement includes:

- how many of the original CPGs included a similar recommendation
- whether the original CPGs recommended or advised against a particular intervention.

Consensus was reached among the author team on the wording of each summary statement to be reflective of the intent of the cluster of recommendations without changing their message (Lomotan et al. 2010). The summary CPG did not intend to describe the strength of the recommendations, rather to provide a user-friendly Tier 3 document that provides clear guidance on the most effective approach to assessment and management for acute and subacute LBP (Grimmer et al. 2019; Machingaidze et al. 2018).

Step 3: The recommendation list was pilot tested on 11 South African physiotherapy clinicians to determine its utility (for instance, acceptability, appropriateness and feasibility) as a Tier 3 document to assist clinical decision-making (Weiner et al. 2017). These clinicians were attending a training programme on CPG use, during which they used the summary recommendations. Most of the clinicians were working in the private sector (*n* = 10). All of the clinicians had limited previous exposure to CPG use in practice. The clinicians resided in a rural town, further limiting their exposure to evidence-based practice courses. The acceptability of intervention measure (AIM), intervention appropriateness measure (IAM) and feasibility of intervention measure (FIM) were used to determine whether it would be a useful document in daily clinical practice (Weiner et al. 2017). Each outcome measure uses a 5-point Likert scale (1 = completely disagree to 5 = completely agree).

### Ethical consideration

Ethical clearance was obtained from the Health Research Ethics Committee of Stellenbosch University, assigned reference number: S17/05/100.

## Results

**FIGURE 1:**
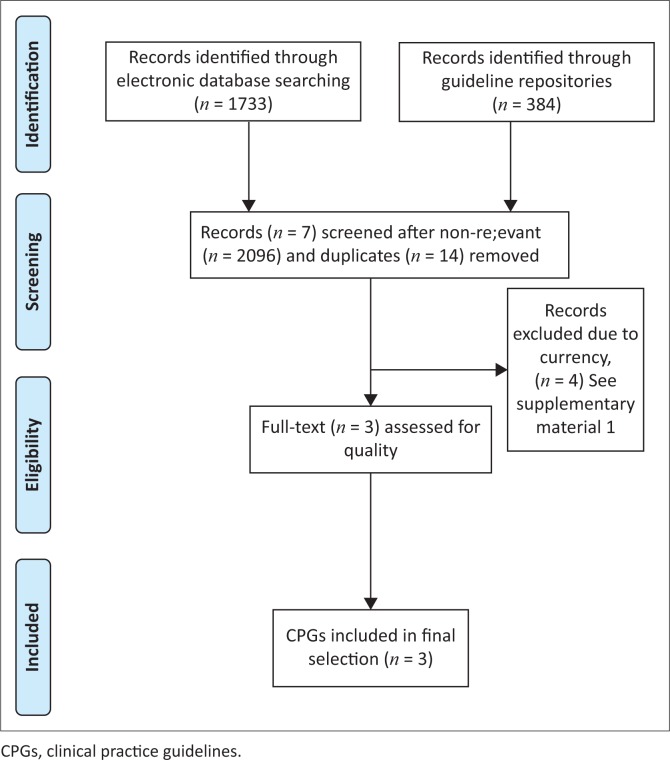
PRISMA flow diagram.

*CPG selection:* Seven potentially relevant CPGs were identified. Figure 1 outlines the PRISMA flow diagram of CPG selection and inclusion (Moher et al. 2009). Four CPGs were excluded because of non-currency (published more than 5 years ago) (Albright et al. 2001; Chou et al. 2007; Delitto et al. 2012; Goertz et al. 2012). The remaining three CPGs (NICE 2016; Qaseem et al. 2017; TOP 2017) were current, and scored well for methodological quality (Supplementary material 1). As all three CPGs were appropriate for use by South African physiotherapists, all relevant recommendations were extracted.

*Draft Tier 3 document:* Box 1 reports 25 summary statements for the assessment and management of acute and subacute LBP. The recommendations were organised under the headings of assessment; red flags; imaging; management; return to work; advice and education; exercise and electrophysical modalities. There was also a ‘For interest only’ section, covering recommendations on blood tests for red flags and pharmacological treatment. This was included for comprehensiveness and to highlight care relevant to multi-disciplinary management. This was printed and laminated and was used during training programme activities to determine the best management approaches for exemplar patients.

BOX 1 Summary of clinical practice guideline.

**Assessment and management of acute and subacute low back pain: Clinical practice guideline summary**

Assessment and management recommendations, extracted verbatim, from three recent, good-quality CPGs:

- Qaseem, A., Wilt, T.J., McLean, R.M. & Forciea, M.A. 2017, ‘Noninvasive treatments for acute, subacute, and chronic low back pain: A clinical practice guideline from the American College of Physicians’, *Annals of Internal Medicine* 166(7), 514–530, viewed from https://annals.org/aim/fullarticle/2603228/noninvasive.
- Toward Optimized Practice (TOP) Low Back Pain Working Group, 2017, *Evidence-informed primary care management of low back pain: Clinical practice guideline*, Toward Optimized Practice, Edmonton, AB, viewed from http://www.topalbertadoctors.org/cpgs/885801.
- National Guideline Centre (Great Britain), 2016, *Low back pain and Sciatica in over 16s: Assessment and management: assessment and non-invasive treatments*, National Institute for Health and Care Excellence, viewed from https://www.nice.org.uk/guidance/ng59.

**Assessment**

All three CPGs recommend that clinicians should conduct a full assessment prior to devising a management plan. The assessment should include history, assessment for red and yellow flags, and a physical assessment. In addition, two CPGs recommend using a risk stratification tool, such as STarT Back, as part of the full assessment.

**Red flags**

All three CPGs recommend identifying patients exhibiting red flags (or specific organic causes) for LBP. These include cancer, infection, trauma, inflammatory disease, etc. An important element of the assessment is therefore to determine if patients have recently developed new or changed symptoms that might indicate organic causes.

All three CPGs recommend referring a patient with suspected red flags for immediate evaluation and management to an appropriate resource, such as a general practitioner, hospital emergency department or medical specialist, depending on what/who is available in a specific setting and local referral protocols.

**Imaging**

All three CPGs recommend that in the absence of red flags, diagnostic imaging (including X-rays, MRI and CT scanning) should not routinely be offered to patients with either acute or subacute LBP.

**Management**

All three CPGs recommend advising the patient to stay active and resume normal activity levels as soon as possible.

All three CPGs recommend using psychological interventions (including cognitive behavioural therapy) to address yellow flags and prevent chronicity after an acute or subacute LBP incident.

Two of the CPGs recommend using spinal mobilisation, manipulation or soft tissue techniques (including massage) for managing patients with acute and subacute LBP. However, one of the CPGs reports that there is insufficient evidence to recommend or to advise against spinal mobilisation.

One CPG recommends that the clinicians should base their management on risk stratification.

Two CPGs advise against offering traction for managing patients with acute and subacute LBP.

One CPG advises against offering belts, corsets, foot orthotics or rocker sole shoes to patients with acute and subacute LBP.

One CPG advises against prescribing bed rest to patients with acute and subacute LBP.

**Return to work**

Two of the CPGs recommend advising the patient to return to work.

**Advice and education**

All three CPGs recommend reassurance of the patient about the general favourable prognosis of patients with acute and subacute LBP, regardless of the management.

**Exercise**

Two of the CPGs recommend advising the patient to start an exercise programme for the management of acute and subacute LBP; however, the type of exercise may vary. Group exercise classes may be of benefit, depending on the patient’s needs and resources available.

**Electrophysical modalities**

Two of the CPGs recommend the prescription of superficial heat, cold or alternating heat and cold (for no longer than 15–20 min) for the home management of patients with acute and subacute LBP.

Two of the CPGs advise against offering ultrasound for the management of patients with acute and subacute LBP.

Two of the CPGs advise against offering transcutaneous electrical nerve stimulation (TENS) for the management of patients with acute and subacute LBP.

Two of the CPGs advise against offering interferential therapy for the management of patients with acute and subacute LBP.

One CPG advises against offering percutaneous electrical nerve stimulation (PENS) for the management of patients with acute and subacute LBP.

There is inconclusive evidence to recommend for or against: acupuncture; back schools; the clinical prediction rule for spinal manipulative therapy; herbal medicine; low-level laser therapy; operant conditioning provided by a physiotherapist; short-wave diathermy and topical NSAIDS

There is insufficient evidence to recommend for or against: craniosacral massage/therapy; modified work duties for facilitating return to work; shock-wave treatment; Tapentadol (Nucynta^®^); touch therapies and yoga therapy

**For interest only**

**Red flags**

Two CPGs recommend referring a patient with red flags for appropriate blood tests if cancer, infection or inflammatory disease is suspected.

**Pharmacologic treatment**

All of the CPGs recommend advising the patient on the use of oral non-steroidal anti-inflammatory drugs for the management of patients with acute and subacute LBP.

Two of the CPGs advise against recommending opioids or steroids for the management of patients with acute and subacute LBP.

There is inconclusive evidence for the use of acetaminophen for the management of patients with acute and subacute LBP.

There is insufficient evidence to recommend for or against analgesic antidepressants, other tricyclic antidepressants, serotonin–norepinephrine reuptake inhibitors (SNRIs) or anticonvulsants for the management of patients with acute and subacute LBP.

CPG, clinical practice guidelines; LBP, low back pain; CT, computed tomography; NSAIDS, non-steroidal anti-inflammatory drugs.

*Tier 3 utility:* The draft Tier 3 document was considered by the participating physiotherapists to be an acceptable, appropriate and feasible end-user document for busy clinicians. Table 2 reports the findings. The acceptability, appropriateness and feasibility of the Tier 3 document scored an average of 4.6 (0.1), 4.6 (0.1) and 4.8 (0.2), respectively, on the instrument’s 5-point Likert scales.

**TABLE 2:** Results of outcome measures.

Outcome measures	P1	P2	P3	P4	P5	P6	P7	P8	P9	P10	P11	Ave
Acceptability of intervention measure AIM	-	-	-	-	-	-	-	-	-	-	-	4.6
1. The summary CPG meets my approval	4	5	4	5	4	4	5	5	5	5	4	4.5
2. The summary CPG is appealing to me	4	5	4	5	4	4	5	5	5	5	4	4.5
3. I like the summary CPG	4	5	4	5	4	4	5	5	5	5	4	4.5
4. I welcome the summary CPG	5	5	4	5	4	4	5	5	5	5	5	4.7
Intervention appropriateness measure IAM	-	-	-	-	-	-	-	-	-	-	-	4.6
1. The summary CPG seems fitting	4	5	4	5	4	4	5	5	5	5	4	4.5
2. The summary CPG seems suitable	5	5	4	5	4	5	5	5	5	5	4	4.7
3. The summary CPG seems applicable	5	5	4	5	4	4	5	5	5	5	4	4.6
4. The summary CPG seems like a good match	5	5	4	5	4	4	5	5	5	5	4	4.6
Feasibility of intervention measure FIM	-	-	-	-	-	-	-	-	-	-	-	4.8
1. The summary CPG seems implementable	4	5	5	4	4	4	5	4	4	5	5	4.5
2. The summary CPG seems possible	5	5	5	4	4	5	5	5	5	5	5	4.8
3. The summary CPG seems doable	5	5	5	5	4	5	5	5	5	5	5	4.9
4. The summary CPG seems easy to use	5	5	5	5	4	5	5	5	4	5	5	4.8

AIM, acceptability of intervention measure; CPG, Clinical practice guidelines; IAM, intervention appropriateness measure; Ave, average.

## Discussion

This article is one of the few articles that outline the development processes of a Tier 3 document, using recommendations from multiple relevant CPGs for one common condition (LBP). The Tier 3 document complemented a physiotherapy training programme about CPG uptake and thus provides new knowledge to assist in evidence implementation for busy clinicians (Machingaidze et al. 2018). Key to the utility of this Tier 3 document was retaining the original recommendations and providing an overall evidence summary for each recommendation cluster. The authors believe that synthesising the recommendations would jeopardise not only the intent of the individual recommendations but also the parent CPGs. There is a growing body of work about how the semantics of writing recommendation from the underpinning research evidence affect its uptake by the target audience (Gupta et al. 2016; Hussain et al. 2009; Shiffman et al. 2005). Whilst recommendations in each cluster appear to use common references, and have similar intent, they often use different words to convey the strength of the body of evidence, and the message. The authors were thus mindful of the difficulties of synthesising the intent of the original recommendation wording and of the potential that a synthesised recommendation would impose different meanings to those of the original recommendations.

The physiotherapists who validated this Tier 3 document believed that it would be a useful quick reference in a busy physiotherapy practice, as it is only two pages in length and is easy to read. Furthermore, they believed that it would be useful to promote evidence-based physiotherapy for LBP to other health care professionals.

The steps that were followed to develop the Tier 3 document could be used to develop similar summary documents for other conditions, as the physiotherapists indicated that this was the type of document they would find easy to access and apply in practice (Stander et al. 2019). Producing Tier 3 documents of organised CPG recommendations from current, high-quality CPGs may be a time- and cost-effective alternative to *de novo* development of CPGs for busy clinicians in a resource-constrained country, such as South Africa (Grimmer et al. 2019; Stander et al. 2019).

The authors acknowledge the limitations of the study. The three parent CPGs did not expand on different approaches to follow with the STarT back risk stratification tool findings (Hill et al. 2011). This may partly be because of the extensive nature of the CPGs, rather than providing step-by-step instructions of individual management approaches. Geographical location, may limit the generalisability of the pilot study’s findings.

## Conclusion

This article presents a step-by-step approach to producing a user-friendly, credible Tier 3 document tailor-made for busy physiotherapy clinicians treating acute and subacute LBP, by using organised recommendations from multiple, relevant CPGs.
